# Altered Splicing of *LAMP2* in a Multigenerational Family from Latvia Affected by Danon Disease

**DOI:** 10.3390/medicina60010099

**Published:** 2024-01-05

**Authors:** Janis Stavusis, Ieva Micule, Ieva Grinfelde, Anna Zdanovica, Janis Pudulis, Sandra Valeina, Svetlana Sepetiene, Baiba Lace, Inna Inashkina

**Affiliations:** 1 Latvian Biomedical Research and Study Centre, Ratsupites 1, LV-1067 Riga, Latvia; 2 Department of Medical Genetics and Prenatal Diagnostics, Children’s University Hospital, Vienibas Gatve 45, LV-1004 Riga, Latvia; 3 Department of Arrhythmology, Riga East University Hospital, Hipokrata 2, LV-1079 Riga, Latvia; 4 Ophthalmology Clinics, Children’s University Hospital, Vienibas Gatve 45, LV-1004 Riga, Latvia

**Keywords:** *LAMP2*, Danon disease, altered splicing

## Abstract

*Background and Objectives*: Danon disease is a multisystemic disorder associated with variants in the *LAMP2* gene, mainly affecting the cardiac muscle. Here, we report a multigenerational family from Latvia with two male patients, hemizygous for a novel splice-affecting variant c.928+3A>G. Affected patients exhibit a cardiac phenotype, moderate mental disability, and mild retinal changes. *Materials and Methods*: Both patients underwent either exome or hypertrophic cardiomyopathy gene panel next-generation sequencing. The pathogenic variant effect was determined using reverse transcription, Sanger sequencing, and high-resolution electrophoresis. *Results*: Evaluation of the splicing process revealed that approximately 80% of the transcripts exhibited a lack of the entire exon 7. This alteration was predicted to cause a shift of the reading frame, consequently introducing a premature stop codon downstream in the sequence. *Conclusions*: Based on our data, we propose that c.928+3A>G is a pathogenic variant associated with Danon disease.

## 1. Introduction

Danon disease, first described by M.J. Danon in 1981 and named after him, is an X-linked dominant multisystemic disease that mainly affects the cardiac muscle, skeletal muscles, and retina [1]. It is also associated with varying levels of cognitive dysfunction, with 70–100% of male patients exhibiting most often mild forms of intellectual disability, with speech and language delays being a common occurrence [2,3,4]. Danon disease has been classified as a lysosomal glycogen storage disease with normal acid maltase activity. It is characterized by the accumulation of intracytoplasmic autophagic vacuoles in cardiac and skeletal muscle fibers [2,5,6].

Similar to most X-linked diseases, males appear to be more severely affected than females, with 100% of affected males having cardiomyopathy, and most male patients dying of heart failure during the second or third decade of life. In contrast, heterozygous pathogenic variants in females can cause late-onset cardiomyopathy and slower disease progression, with an overall milder phenotype [4,5,7]. Some females, however, experience early disease onset and more severe progression [8]. Interestingly, most male patients exhibit hypertrophic cardiomyopathy, whereas roughly 50% of female patients exhibit dilated cardiomyopathy [9]. A retinal phenotype is also mainly observed in male patients, with 69% having pathologic changes centering around the loss of pigment [4].

The functional phenotypes of patients with Danon disease have been associated with heterozygous and hemizygous variants in the *LAMP2* gene [7,10,11,12]. Most disease-causing variants are nonsense or frameshift alterations, resulting in the premature termination of the encoded lysosome-associated membrane protein-2 (LAMP2). These proteins are usually missing the transmembrane and cytoplasmic domains of full-length LAMP2, with Western blot analysis of muscle biopsies showing a marked decrease or complete absence of the protein [4,10]. Variants that affect the splicing of the gene have also been associated with variable features of Danon disease in both males and females, along with decreased expression of the encoded protein [12,13,14].

The *LAMP2* gene is located on the X chromosome at locus q24 and consists of nine exons. It encodes LAMP2, a 410-amino acid protein consisting of a heavily glycosylated luminal domain, a transmembrane domain, and a short C-terminal cytoplasmic tail, containing a lysosomal-targeting signal [5]. The protein is located in the membrane of vesicles, with the combination of LAMP2 and LAMP1 constituting a significant fraction of all lysosomal membrane glycoproteins [15]. Alternative splicing of the last exon of *LAMP2*, which encodes part of the luminal, transmembrane, and cytoplasmic domains, yields three different LAMP2 isoforms, LAMP-2a, LAMP-2b, and LAMP-2c, which differ in subcellular localization and tissue distribution patterns [16].

The LAMP-2a isoform is well researched, and although it is widely expressed in various tissues, its expression is low in skeletal muscles and the brain [17,18]. LAMP-2a protects lysosomal membranes, which contain proteolytic enzymes, such as acid hydrolases responsible for the degradation of foreign materials and specialized autolytic functions. The isoform plays a major role in chaperone-mediated autophagy by binding to target proteins and targeting them for lysosomal degradation [19,20,21]. Disruption of chaperone-mediated autophagy is associated with various pathologic conditions, including cardiovascular and neurodegenerative diseases and cancer [22]. LAMP-2a is also required for the fusion of autophagosomes with lysosomes during autophagy, resulting in the degradation of the contents of autophagosomes [23].

In addition to a wide low-level expression, the LAMP-2b isoform is highly expressed in human skeletal muscles, the heart, and the brain [17,24]. The main function of the LAMP-2b isoform is thought to be the regulation of lysosome maturation, and the lack of this isoform is associated with Danon disease [10].

The final isoform LAMP-2c is expressed only in the small intestine, heart, brain, and skeletal muscle. It is associated with nucleic acid degradation and the presentation of cytoplasmic antigens [18,22].

## 2. Objective

The present study describes a multigenerational family from Latvia with Danon disease, carrying a previously undescribed intronic variant, c.928+3A>G, in the *LAMP2* gene.

## 3. Patients

**Figure 1:**
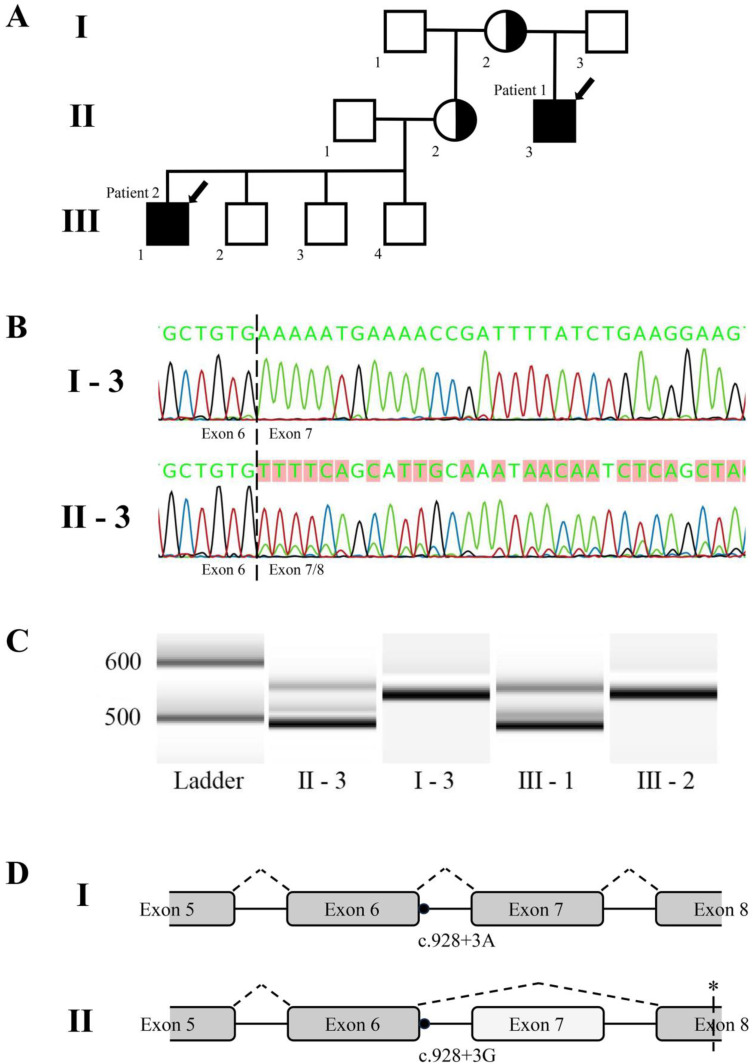
(**A**) Family pedigree, indicating Patient 1 (II-3) and Patient 2 (III-1), as well as both heterozygous carriers (II-2 and I-2). Black or white shading indicates affected and unaffected individuals, respectively, while index patients are denoted with arrows. Individuals shaded in black and white indicate confirmed heterozygotes. (**B**) Sequencing results of *LAMP2* transcripts from a patient (panel **B**, **bottom**) and a healthy relative (panel **B**, **top**), covering the boundary of exons 6 and 7 (indicated by the dashed line). In the affected patient sample, after the splice site, the predominant sequence is from exon 8, with the intact sequence of exon 7 in the background. (**C**) Bioanalyzer-based imaging of a PCR fragment spanning exons 5 to 9, expected to be 538 bp in length in a wild-type transcript. Patient samples used for amplification are denoted below the gel bands. (**D**) A schematic representation of the observed changes in RNA splicing—the omission of exon 7. The dot represents the variant location, with the corresponding nucleotide at the given location denoted below. The asterisk represents the location of the predicted premature stop codon.

The multi-generational family described in this study includes two patients affected with Danon disease (MIM#300257, Figure 1A). Both patients were recruited in the Genome Database of Latvian Population (Riga, Latvia) in the framework of the ERDF research project “The determination of rare inherited diseases’ causative mechanisms using whole genome sequencing approach”. Data and biological material were collected from members of this family with approval from the Central Medical Ethics Committee of Latvia (protocol No. 2019-3, chapter 7, from 30 May 2019), which covers all consent- and data handling-related issues for genetic research of the patients involved. All participants or their parents provided written informed consent.

Patient 1 (II-3) is a 22-year-old male. His early development was unremarkable: he started walking at 14 months and developing language at two years of age. However, a learning disability was suspected when he started primary school. The first formal IQ test, administered at the age of 15, yielded a score of 50, consistent with a moderate mental disability. At the age of 16, he presented at a cardiology department with a paroxysm of supraventricular tachycardia after some physical activity. Subsequent examination revealed hypertrophic cardiomyopathy with hypertrophy of the left ventricle but with a retained cardiac function of 60%, Wolff–Parkinson–White (WPW) syndrome, and second-grade hypertension. Additional findings included adiposity (BMI 29 kg/m^2^), elevated liver enzymes, low-density lipoprotein cholesterol (LDL-C), and latent hypothyroidism. His creatine kinase levels were at the upper limit of the reference range, 175–297 U/L (Ref < 190 U/L), and he showed no evidence of proximal muscular weakness. Ophthalmological examination showed non-specific cystic changes in the macular region and tigroid pattern changes in the periphery, but he had no complaints about vision (Supplemental Figure S1). During a recent visit to the cardiologist, the patient reported frequent fluctuations in blood pressure and diminished exercise tolerance. Betaxolol and Perindopril indapamide combined therapy abolished the recurrence of the supraventricular tachycardia. The electrocardiogram depicted a sinus rhythm of 76 bpm, left ventricular hypertrophy with secondary ST and T changes, indications of WPW syndrome, and one premature atrial complex with extreme preexcitation. Holter electrocardiogram findings indicated a mean heart rate of 67 bpm, corroborated signs of WPW syndrome, and revealed premature atrial complexes, as well as several atrial couplets, atrial runs, and greatly prolonged QRS complexes with a frequency of 130 bpm. Echocardiography disclosed a markedly increased thickness of the posterior wall (15–20 mm) and the interventricular septum (16–20 mm), particularly in the apical segments, reminiscent of observations in patients with Yamaguchi syndrome. The left ventricular ejection fraction was preserved at 70%, with no conclusive evidence of left ventricular outflow tract obstruction or pulmonary hypertension. The need for an implantable cardioverter defibrillator was not indicated, and the patient refused both electrophysiology study and catheter ablation for the WPW syndrome.

Patient 2 (III-1) is a 17-year-old male with unremarkable early motor development: he started walking at 14 months and had a mild language delay, with language first developing at the age of three. The patient underwent corrective surgery for an atrioventricular septal defect (AVSD) at the age of four. His psychomotor development was delayed, with the patient experiencing epileptic seizures starting at the age of seven. The patient attends a specialized school, at which an episode of physical activity led to a loss of consciousness and resulted in the finding of hypertrophic cardiomyopathy. Adiposity was also detected, as were mildly but inconsistently elevated creatine kinase levels, ranging from 86 to 246 U/L. Currently, the patient is intolerant to physical exertion and exhibits traits consistent with moderate mental disability, but he has not been formally tested. Patient 2 has no complaints about his vision, and an ophthalmological examination revealed no retinal changes.

The mother of patient 1 (I-2) is currently 65 years old. During her last visit to the cardiologist, she had no cardiac-related complaints, while her medical history entailed unspecified hypothyroidism, arterial hypertension, and dyslipidemia. Her electrocardiogram showed a sinus rhythm of 64 bpm, one premature ventricular complex, one atrial couplet, and unspecified intraventricular and T-wave changes. Echocardiography revealed mild left ventricular hypertrophy, with a wall thickness of up to 11 mm, but a preserved left and right ventricular ejection fraction and no evidence of valve abnormalities or pulmonary hypertension.

The mother of patient 2 (II-2) is currently 41 years old. She has complaints of dyspnea and fatigue. Her electrocardiogram shows high voltage, an ectopic atrial rhythm of 57–62 bpm, periodic bradycardia, frequent supraventricular extrasystoles (including trigeminy), and some AV connection extrasystoles. Her past echocardiography revealed a thickened cardiac wall, but it has been several years since she last sought consultation from a cardiologist.

None of the mothers has a clinically diagnosed intellectual disorder; however, none of them have undergone formal cognitive testing. For patient phenotype summary, see Table 1.

## 4. Methods

### 4.1. DNA Extraction and Next-Generation Sequencing

Total DNA was extracted from peripheral blood leukocytes using the standard phenol/chloroform extraction method. Both patients underwent either exome or hypertrophic cardiomyopathy (HCM) gene panel sequencing, with tests performed by a private company. In short, the samples were processed and sequenced using the Illumina platform (San Diego, CA, USA) and a hybridization-based enrichment protocol, with median coverage depths of 150× and 190× for exome and panel sequencing, respectively. The obtained raw sequencing reads were mapped to the human reference genome (GRCh37/hg19). Burrows–Wheeler Aligner (BWA-MEM) software (https://bio-bwa.sourceforge.net/ (accessed on 23 November 2023)) (Cambridge, UK) was used for read alignment. The fastq files were processed and variants were called using GATK v4 (Cambridge, MA, USA) best practice guidelines. Variant filtration was conducted, focusing on rare variants in coding regions, exon–intron boundaries (±20 bps), and selected non-coding, deep intronic variants predicted to affect the protein function for the known disease-associated genes. The company provided any such variants in the final test report. All reported sequence changes were manually curated, and all clinically significant observations were confirmed by Sanger sequencing.

### 4.2. RNA Extraction, cDNA Synthesis, and Sanger Sequencing

RNA was extracted from peripheral blood using the RNeasy Mini Kit (Qiagen, Hilden, Germany). Reverse transcription was conducted using the RevertAid First Strand cDNA Synthesis Kit (ThermoFisher Scientific, Waltham, MA, USA) with random hexamer primers. The cDNA samples were subjected to region-specific amplification, targeting exons 5 to 8 of the *LAMP2* gene, using the HOT FIREPol polymerase (Solis Biodyne, Tartu, Estonia) and custom primers (sequences available upon request). To obtain a quantitative representation of the amplified transcript ratio, high-resolution electrophoresis was performed using the Bioanalyzer 2100 system (Agilent, Santa Clara, CA, USA) and the High Sensitivity DNA Kit (Agilent, Santa Clara, CA, USA).

Amplification products were subjected to Sanger sequencing using BigDye™ Terminator v3.1 Cycle Sequencing Kit (ThermoFisher Scientific, Waltham, MA, USA) and an ABI PRISM 3100 genetic analyzer (Applied Biosystems, Waltham, MA, USA).

## 5. Results

The genetic testing of both affected individuals identified a single intronic variant, c.928+3A>G, in the disease-associated *LAMP2* gene (here and further RefSeq No. NM_002294.3, variant submitted to the ClinVar database (Accession#VCV001380172.3)), which was the only one that co-segregated with the disease. At the time of testing, this variant was previously undescribed and classified as a variant of unknown significance, as in silico tools did not predict a significant impact on splicing (SSF, MaxEntScan, NNSPLICE). The site is, however, genetically conserved in mammals, with a PyloP100 score of 4.6 and a PhastCons score of 1.0. Subsequent use of the VarSome engine, based on ACMG guidelines, identified this variant as likely pathogenic because it is located close to a canonical splice site and the position is conserved; furthermore, it had not yet been reported in the gnomAD database.

To evaluate the effects of the c.928+3A>G variant on *LAMP2* mRNA splicing, RNA was extracted from peripheral blood samples of both patients and their closest unaffected family members, followed by cDNA generation and region-specific amplification. The results revealed that roughly 80% of the transcripts of the amplified region in the affected patients’ samples lacked all of exon 7 (64 bp in length), with the remaining 20% retaining the complete correct sequence (Figure 1B,C).

No changes in transcripts, however, were observed in the samples from unaffected family members. The loss of exon 7 would cause a shift in the open reading frame and introduce a premature stop codon downstream (Figure 1D).

After disease confirmation in the two patients, DNA samples from both mothers were tested in a clinical laboratory, and it was proven that the females were heterozygous carriers of the causative variant. Unfortunately, no female carrier blood samples could be obtained for RNA extraction and splicing analysis.

## 6. Discussion

Variants affecting splice sites in the *LAMP2* gene have been previously associated with disease phenotypes [12,13,14]. For example, members of a large family carrying the c.864+4A>G variant presented with cardiomyopathy and most had arrhythmia, but they varied in behavioral problems, mental disability, and skeletal myopathy [12]. Evaluation of a family carrying the c.741+2T>C variant found that the affected individuals exhibited only cardiac-related symptoms, such as ventricular tachycardia, intraventricular block, and hypertrophic cardiomyopathy [13]. A familial case of the c.864+5G>A variant has also been described, resulting in skipping exon 6 but not introducing a shift of the reading frame, and presenting mainly with hypertrophic cardiomyopathy [14]. 

In comparison, our male patients show similar cognitive and cardiac phenotypes (hypertrophic cardiomyopathy) to the ones described; furthermore, both of them lack the skeletal features of Danon disease as well. However, patient 2 exhibits additional features that are not characteristic of Danon disease—epilepsy and a congenital heart defect. It is possible that the etiology of these symptoms could be unrelated to the *LAMP2* variant. In contrast, the heterozygous females from this pedigree show quite discordant cardiac phenotypes, with the youngest (II-2, 41 years old) exhibiting more pronounced changes on an electrocardiogram and having clinical complaints, while the older (I-2, 65 years old) reports no cardiac complaints and shows only minimal changes in cardiac investigations, which could be associated with arterial hypertension.

Evaluation of the family in the present study showed that the splice site variant c.928+3A>G resulted in a *LAMP2* gene transcript lacking exon 7. The lack of this exon was predicted to introduce a frameshift and a premature stop codon downstream, thus likely encoding a non-functional or degraded protein. We believe the altered splicing could be considered to be criterion PS3 (strong evidence) according to ACMG guidelines, which therefore warrants the change of the classification of this variant to pathogenic. Since our initial uploading of the variant to the ClinVar database, it has been reported once more by the commercial genetic testing company Invitae. In this submission, it has been classified as a variant of unknown significance, probably owing to it not being tested beyond next-generation sequencing. Because of this, the variant has now been classified in ClinVar as a variant with conflicting interpretations of pathogenicity. Furthermore, another variant in the same position (c.928+3A>T) was described by Bournazos et al. in 2022 [25]. This variant has been classified as pathogenic by ClinVar and likely pathogenic by the Varsome platform. There is no extensive description of affected patients in this publication, but the evidence provided shows the exclusion of the entire exon 7, similar to our findings. Interestingly, a complete lack of exon was demonstrated in their samples, while our results show roughly 20% of the full-length *LAMP2* transcript remaining [25]. The mechanism involved is unclear, although it may be due to an as-yet unidentified splicing variant or a splice site that is not working with 100% efficiency.

Due to the three known LAMP2 protein isoforms sharing the affected region, the c.928+3A>G variant likely affects the functionality of all. Undoubtedly, electron microscopy, immunohistochemical staining, and Western blotting assessment of LAMP2 protein levels and localization in patient biopsy samples are required to fully characterize this novel variant. However, no such samples are currently available, and neither the primary patients nor the heterozygous carriers have agreed to perform biopsies. 

Interestingly, the two patients were initially examined by two different geneticists. The relatedness of the two patients was confirmed only after the results of genetic testing were received, indicating that independent evaluations of the two patients resulted in similar conclusions.

## Figures and Tables

**Table 1:** Summary of patient clinical features. N/A—no data available or no clinical evaluation performed.

Patient	Variant Status	Cardiac Symptoms	Cognitive Impairment	Retinal Symptoms	Myopathic Symptoms
Patient 1 (II-3)	c.928+3A>G/−	Hypertrophic cardiomyopathy with hypertrophy of the left ventricle. Wolff–Parkinson–White syndrome. Second-grade hypertension.	Moderate mental disability.	Non-specific cystic changes in the macular region and tigroid pattern changes in the periphery.	None
Patient 2 (III-1)	c.928+3A>G/−	Hypertrophic cardiomyopathy. Atrioventricular septal defect.	Moderate mental disability. Epileptic seizures.	None	None
Mother of patient 1 (I-2)	c.928+3A>G/WT	Minimal changes.	N/A	N/A	N/A
Mother of patient 2 (II-2)	c.928+3A>G/WT	Heart rate and rhythm regulation problems.	N/A	N/A	N/A

## Data Availability

The data presented in this study are openly available in ClinVar database at: https://www.ncbi.nlm.nih.gov/clinvar, reference number VCV001380172.3.

## Supplementary Materials

The following supporting information can be downloaded at: https://www.mdpi.com/article/10.3390/medicina60010099/s1, Supplemental Figure S1: Ocular fundus images of Patient 1 illustrate non-specific cystic changes in the macular region and tigroid pattern changes in the periphery.

## References

[1] Danon M.J., Oh S.J., DiMauro S., Manaligod J.R., Eastwood A., Naidu S., Schliselfeld L.H. (1981). Lysosomal glycogen storage disease with normal acid maltase. Neurology.

[2] Roseline F., Irène M. Orphanet: Glycogen Storage Disease Due to LAMP 2 Deficiency. https://www.orpha.net/consor/cgi-bin/OC_Exp.php?Lng=GB&Expert=34587.

[3] Taylor M.R., Adler E.D. (2020). Danon Disease. GeneReviews^®^.

[4] D’Souza R.S., Levandowski C., Slavov D., Graw S.L., Allen L.A., Adler E., Mestroni L., Taylor M.R.G. (2014). Danon disease: Clinical features, evaluation, and management. Circ. Heart Fail..

[5] Hong D., Shi Z., Zhang W., Wang Z., Yuan Y. (2012). Danon disease caused by two novel mutations of the LAMP2 gene: Implications for two ends of the clinical spectrum. Clin. Neuropathol..

[6] Sugie K., Noguchi S., Kozuka Y., Arikawa-Hirasawa E., Tanaka M., Yan C., Saftig P., von Figura K., Hirano M., Ueno S. (2005). Autophagic vacuoles with sarcolemmal features delineate Danon disease and related myopathies. J. Neuropathol. Exp. Neurol..

[7] Arad M., Maron B.J., Gorham J.M., Johnson W.H.J., Saul J.P., Perez-Atayde A.R., Spirito P., Wright G.B., Kanter R.J., Seidman C.E. (2009). Glycogen Storage Diseases Presenting as Hypertrophic Cardiomyopathy. N. Engl. J. Med..

[8] Hedberg Oldfors C., Máthé G., Thomson K., Tulinius M., Karason K., Östman-Smith I., Oldfors A. (2015). Early onset cardiomyopathy in females with Danon disease. Neuromuscul. Disord..

[9] Yang Z., McMahon C.J., Smith L.R., Bersola J., Adesina A.M., Breinholt J.P., Kearney D.L., Dreyer W.J., Denfield S.W., Price J.F. (2005). Danon disease as an underrecognized cause of hypertrophic cardiomyopathy in children. Circulation.

[10] Nishino I., Fu J., Tanji K., Yamada T., Shimojo S., Koori T., Mora M., Riggs J.E., Oh S.J., Koga Y. (2000). Primary LAMP-2 deficiency causes X-linked vacuolar cardiomyopathy and myopathy (Danon disease). Nature.

[11] Charron P., Villard E., Sébillon P., Laforêt P., Maisonobe T., Duboscq-Bidot L., Romero N., Drouin-Garraud V., Frébourg T., Richard P. (2004). Danon’s disease as a cause of hypertrophic cardiomyopathy: A systematic survey. Heart.

[12] Roos J.C.P., Daniels M.J., Morris E., Hyry H.I., Cox T.M. (2018). Heterogeneity in a large pedigree with Danon disease: Implications for pathogenesis and management. Mol. Genet. Metab..

[13] Li Z., Ma F., Li R., Xiao Z., Zeng H., Wang D.W. (2021). Case Report: A Novel *LAMP2* Splice-Altering Mutation Causes Cardiac-Only Danon Disease. Front. Cardiovasc. Med..

[14] Fu D., Wang S., Luo Y., Wu S., Peng D. (2023). Identification of a novel splicing-altering *LAMP2* variant in a Chinese family with Danon disease. ESC Heart Fail..

[15] Fukuda M., Viitala J., Matteson J., Carlsson S.R. (1988). Cloning of cDNAs encoding human lysosomal membrane glycoproteins, h-lamp-1 and h-lamp-2. Comparison of their deduced amino acid sequences. J. Biol. Chem..

[16] Eskelinen E.L., Cuervo A.M., Taylor M.R.G., Nishino I., Blum J.S., Dice J.F., Sandoval I.V., Lippincott-Schwartz J., August J.T., Saftig P. (2005). Unifying nomenclature for the isoforms of the lysosomal membrane protein LAMP-2. Traffic.

[17] Konecki D.S., Foetisch K., Zimmer K.P., Schlotter M., Konecki U.L. (1995). An alternatively spliced form of the human lysosome-associated membrane protein-2 gene is expressed in a tissue-specific manner. Biochem. Biophys. Res. Commun..

[18] Pérez L., McLetchie S., Gardiner G.J., Deffit S.N., Zhou D., Blum J.S. (2016). LAMP-2C Inhibits MHC Class II Presentation of Cytoplasmic Antigens by Disrupting Chaperone-Mediated Autophagy. J. Immunol..

[19] Fukuda M. (1994). Biogenesis of the lysosomal membrane. Subcell Biochem.

[20] Cuervo A.M., Dice J.F. (2000). Unique properties of lamp2a compared to other lamp2 isoforms. J. Cell Sci..

[21] Bandyopadhyay U., Kaushik S., Varticovski L., Cuervo A.M. (2008). The chaperone-mediated autophagy receptor organizes in dynamic protein complexes at the lysosomal membrane. Mol. Cell. Biol..

[22] Qiao L., Hu J., Qiu X., Wang C., Peng J., Zhang C., Zhang M., Lu H., Chen W. (2023). LAMP2A, LAMP2B and LAMP2C: Similar structures, divergent roles. Autophagy.

[23] Hubert V., Peschel A., Langer B., Gröger M., Rees A., Kain R. (2016). LAMP-2 is required for incorporating syntaxin-17 into autophagosomes and for their fusion with lysosomes. Biol. Open.

[24] Rowland T.J., Sweet M.E., Mestroni L. (2016). Taylor MRG. Danon disease—Dysregulation of autophagy in a multisystem disorder with cardiomyopathy. J. Cell Sci..

[25] Bournazos A.M., Riley L.G., Bommireddipalli S., Ades L., Akesson L.S., Al-Shinnag M., Alexander S.I., Archibald A.D., Balasubramaniam S., Berman Y. (2022). Standardized practices for RNA diagnostics using clinically accessible specimens reclassifies 75% of putative splicing variants. Genet. Med..

