# Supplementary figures and images for: Altered Splicing of LAMP2 in a Multigenerational Family from Latvia Affected by Danon Disease
Source: Medicina (Kaunas). 2024 Jan 5;60(1):99. doi: 10.3390/medicina60010099 (PMC10821070; doi:10.3390/medicina60010099)

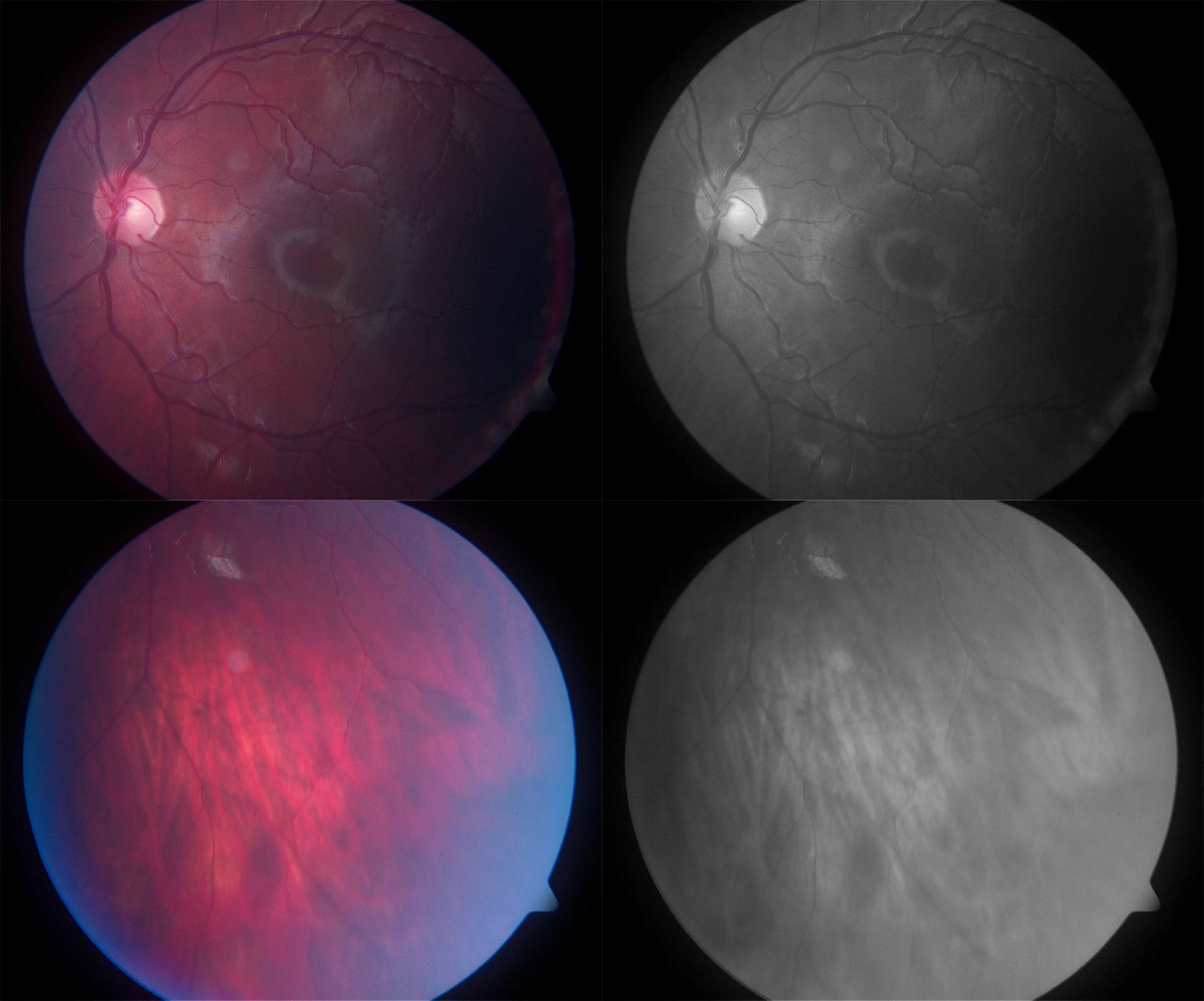

Supplement: Supplementary file 1 [file medicina-60-00099-s001.zip › medicina-2763491-supplementary.tif]
